# Effects of heat–moisture, autoclaving, and microwave treatments on physicochemical properties of proso millet starch

**DOI:** 10.1002/fsn3.1295

**Published:** 2020-01-13

**Authors:** Ming‐zhu Zheng, Yu Xiao, Shuang Yang, Hui‐min Liu, Mei‐hong Liu, Sanabil Yaqoob, Xiu‐ying Xu, Jing‐sheng Liu

**Affiliations:** ^1^ College of Food Science and Engineering Jilin Agricultural University Changchun Jilin China; ^2^ National Engineering Laboratory for Wheat and Corn Deep Processing Changchun Jilin China; ^3^ College of Life Science Jilin Agricultural University Changchun Jilin China

**Keywords:** autoclaving treatment, heat–moisture treatment, microwave treatment, proso millet starch

## Abstract

Proso millet starch was modified by heat–moisture treatment (HMT), autoclaving treatment (AT), and microwave treatment (MT). The effects of these treatments on the starch physicochemical, structural, and molecular properties were investigated. The amylose and resistant starch contents were increased by AT and MT, but only slightly by HMT. HMT and AT significantly increased the water‐holding capacity, to 172.66% and 191.63%, respectively. X‐ray diffractometry showed that the relative crystallinity of the HMT sample decreased by 20.88%, and the crystalline peaks disappeared from the AT and MT sample patterns. The thermal treatments decreased the proso millet starch molecular weight to 1.769 × 10^6^, 7.886 × 10^5^, and 3.411 × 10^4^ g/mol, respectively. The thermal enthalpy decreased significantly in HMT. Modification significantly changed the pasting profiles of the native proso millet starch, and the peak viscosity, setback, and breakdown values decreased. These results clarify the mechanism of starch changes caused by thermal treatment.

## Graphical abstract

In this study, heat–moisture, autoclaving, and microwave treatments affect the physicochemical properties of proso millet starch, and microwave treatment has a greater impact on proso millet starch.

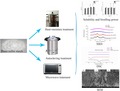

## 1 INTRODUCTION

Proso millet (*Panicum miliaceum *L.) has excellent nutritional properties and is a fundamental resource in crop‐breeding programs and food diversification (Cho et al., 2010). It is considered to be a new medical food homologous crop and is rich in protein, starch, dietary fiber, and a variety of trace elements such as Mg, Fe, and Ca. It also can prevent arteriosclerosis, gastrointestinal cancer, coronary heart disease, and other diseases (Saleh, Zhang, Chen, & Shen, 2013). Interest in millet use has increased because of various rediscovered health benefits and especially in food applications as a nongluten ingredient (Fan, 2014). Proso millet is considered to be an underused grain in China. It contains abundant starch, with a reported content 60%–70%. Because of the extensive use of starch in food systems, different sources with good functional properties are being explored (Rose & Santra, 2013).

Starch has a wide range of applications in the food industry. It is used for thickening, gelling, and stabilization and can replace expensive raw materials, which expands its application range (Radley, 1976). Increasing attention is being paid to starch because of its wide applicability, low cost, and specific functions. However, methods commercial methods for processing native starch need to be improved to extend the product shelf life (French, 1984). A typical process generally leads to viscosity reduction, structural loosing, and product deterioration under long‐term storage conditions, especially in repeated freezing treatments.

Heat–moisture treatment (HMT) of starches refers to exposure of starch granules at a temperature above the glass transition temperature but below the gelatinization temperature for a certain time period and at restricted moisture content. HMT causes physical modifications of the starch granules with respect to size, shape, and birefringence (Hoover & Manuel, 1996). Autoclaving treatment (AT) promotes hydration of the amorphous zone in starch granules under the action of a pressure field. The amorphous layer of the starch granule crystallization zone swells in water with increasing pressure, which causes rearrangement of the amylopectin double helices (Bravo, Siddhuraju, & Saura‐Calixto, 1998). Microwave treatment (MT) affects starch through dielectric heating and electromagnetic polarization effects (Bilbao‐Sáinz, Butler, Weaver, & Bent, 2007). Modification alters the starch physicochemical properties and can improve the functionality of native starch (Bemiller & Huber, 2015). Previously reported studies of proso millet starch have focused on crop breeding and genotype (Wang et al., 2017), or comparing the physicochemical properties of different varieties (Wang et al., 2018). Less work has been done on the effects of HMT, AT, and MT on the physicochemical properties of proso millet starch. The proximate composition of proso millet is similar to those of other common grains (e.g., rice, corn, and wheat) (Park et al., 2018; Silva et al., 2017; Zhao et al., 2018), and understanding the processing properties of proso millet starch would be conducive to its development and use.

The aim of this study was to explore the behaviors of native proso millet starch and starches modified by different thermal treatments that could improve the value of proso millet starch and proso millet in the food industry. This study will clarify the mechanisms involved in thermal treatments of starch and enable full use of proso millet resources.

## 2 MATERIALS AND METHODS

### 2.1 Materials

Proso millet starch with an amylose content of 13% was kindly supplied by the National Engineering Laboratory for Wheat and Corn Deep Processing, China. All chemicals were analytical grade and used as obtained, without further purification.

### 2.2 Sample preparation

Proso millet starch (20 g, dry basis) and water (80 ml) were mixed well. The HMT sample was heated for 30 min at 100°C in a sealed glass bottle, the AT sample was heated for 30 min at 121°C (0.1 MPa), and the MT sample was heated at a microwave power of 500 W for 10 min. The samples were then freeze‐dried, ground, and stored in plastic vials until further use.

### 2.3 **Amylose**, **amylopectin**, **and resistant starch content**

Amylose/amylopectin assay with K‐AMYL 07/11 (Megazyme International Ireland, Ltd.) was used for amylose and amylopectin determination; the method described by Gibson, Solah, and Mccleary (1997) was used.

A K‐RSTAR kit (Megazyme International Ireland Ltd.) was used to determine the resistant starch content. The samples were hydrolyzed with α‐amylase and amyloglucosidase (provided with the kit) for 16 hr at 37°C. In all the methods, free glucose formed by enzymatic hydrolysis was quantified colorimetrically with an oxidase–peroxidase glucose reagent.

### 2.4 **Water**‐**holding capacity**

The Water holding capacity (WHC) was determined by the method described by Yamazaki (1953). The sample (2 g, dry basis) was dissolved in distilled water (25 ml) by shock mixing, soaked at room temperature for 30 min to form a uniform paste, and centrifuged (2,000 *g*, 10 min). After separation of the supernatant, the water content of the sediment was determined directly by weighing.

### 2.5 Solubility and swelling power

The solubility and swelling power were determined by using a modified version of the leaching method reported by Adebowale, Afolabi, and Olu‐Owolabi (2005). Starch (0.2 g) was dissolved in water (10 ml). The samples were subjected to intermittent shocks in a water bath for 30 min at 60, 70, 80, and 90°C. The treated samples were cooled to room temperature and then centrifuged at 2,000 *g* for 15 min. The supernatant was poured into an aluminum box and dried at 105°C to obtain the water‐soluble starch. The precipitate was swelled starch.

(1) $$\text{Solubility}\;\left(\%\right)=\frac{W_{\text{ss}}}{W_{s}}\times 100\%,$$

(2) $$\text{Swelling}\;\text{power}\;\left(\%\right)=\frac{W_{\text{sp}}}{W_{s}\left(100\%-\text{solubility}\right)}\times 100\%,$$

where *W*_ss_ is the weight of soluble starch (g), *W*_sp_ is the weight of sediment paste (g), and *W*_s_ is the weight of the sample (g).

### 2.6 HPSEC‐MALLS‐RI

The weight‐average molecular weight (*M*_w_), z‐average radius of gyration (*R*_z_), and polydispersity index (PDI) of the samples were determined by high‐performance size‐exclusion chromatography coupled with multiangle laser‐light scattering and refractive index detection (HPSEC‐MALLS‐RI) under the conditions described by Zhang, Li, Chen, and Situ (2016) with some modifications. Starch (50 mg) was dispersed in dimethyl sulfoxide (DMSO; 2 ml). The suspension was heated in a boiling‐water bath for 15 min with intermittent stirring. Aqueous ethanol (95%, v/v, 6 ml) was added to the starch suspension to precipitate the starch. After standing for 15 min, the ethanol‐precipitated starch was separated by centrifugation at 3,000 *g* for 10 min, the supernatant was discarded, and the tubes were drained on tissue paper for 15 min. The starch pellet was redissolved in DMSO (90%, v/v, 5 ml) with LiBr (50 mmol/L) and left at 60°C overnight. Before injection, the sample solution was filtered through a membrane filter (5.0 μm). The mobile phase was DMSO (HPLC grade, 90%, v/v) with LiBr (50 mmol/L) and was filtered through a 0.22‐μm membrane filter and degassed by ultrasound before use; the injection volume was 100 μl. The flow rate was 0.5 ml/min, the column was maintained at 60°C, and the *d_n_*/*d_c_* value was 0.0740 ml/g. The data were analyzed by Astra software (Wyatt Technology).

### 2.7 X‐ray diffractometry

The crystal structures of the native and modified samples were investigated by X‐ray diffractometry (XRD) (Rigaku AXS Model SmartLab) under the conditions described by Watcharatewinkul, Puttanlek, Rungsardthong, and Uttapap (2009). RD patterns were recorded at 40 kV and 40 mA with Cu Kα radiation (*λ* = 0.15405 nm). Scanning was performed from 3° to 50° (2*θ*) with a step interval of 0.02° and a scanning rate of 10°/min. The relative crystallinity was calculated as the ratio of the crystalline peak area to the total diffraction area.

### 2.8 Differential scanning calorimetry

All Differential scanning calorimetry (DSC) data were obtained with a Q2000 instrument (TA, USA). The sample processing method described by Hélène et al. (2007) was used. Briefly, samples were prepared in triplicate, by accurately weighing starch (2 mg) and dissolving it in deionized water (10 ml) in a pan, mixing, and holding for 12 hr to equilibrate. Samples were heated from 40 to 120°C at a rate of 10°C/min. An empty aluminum pan was used as the control.

### 2.9 Pasting properties

The pasting properties of the samples were determined with a Rapid Visco Analyser (Model RVA; Newport Scientific) by using a standard starch profile. A mixture of the sample (3.5 g) in distilled water (25 ml) was stirred at 160 rpm. The samples were held at 50°C for 1 min and then heated to 95°C at 4°C/min and then held at 50°C for 5 min. The pasting temperature, peak viscosity (PV), breakdown (BD) value, final viscosity (FV), and setback (SB) value were recorded.

### 2.10 Scanning electron microscopy

The sample particle microstructures were examined by Scanning electron microscopy (SEM) (PW‐100‐011; LASER Company); the method described by Kiseleva et al. (2005) was used. Freeze‐dried samples were mounted on an aluminum stub by using double‐sided sticky tape and coated with a thin film of gold. Images were recorded at an accelerating voltage of 20 kV.

### 2.11 Statistical analysis

Sample analyses were performed in triplicate, and standard deviations were reported. A comparison of the means was ascertained by Tukey's test to be at a 5% level of significance by analysis of variance.

## 3 RESULTS AND DISCUSSION

### 3.1 Amylose, amylopectin, and resistant starch contents

The amylose, amylopectin, and resistant starch contents of proso millet starch samples modified by three different methods are presented in Table 1. The amylose contents of the HMT, AT, and MT samples increased to 16.3%, 18.7%, and 22.1%, respectively, and the resistant starch contents increased to 12.3%, 15.5%, and 18.7%, respectively. When starch is subjected to gelatinization, the molecules align and some of the amylopectin is hydrolyzed to short amylose. The heat transfer efficiencies for HMT, AT, and MT were different. HMT partially decomposed the amylopectin in the starch by gelatinization. For MT, microwave gelatinization can destroy the starch crystallinity before particle expansion, and therefore, more of the molecular material was leached at higher temperatures (Palav & Seetharaman, 2006). Gelatinization under pressure may promote fracture of the amylopectin molecular structure or hydrolysis of long‐chain amylose molecules to short‐chain molecules.

**Table 1:** The amylose, amylopectin, and resistant starch content of native proso millet starch, HMT, AT, and MT starches

Type	Amylose (%)	Amylopectin (%)	Resistant starch (%)
N	14.7 ± 1.2^d^	83.1 ± 0.8^a^	11.5 ± 0.3^c^
HMT	16.3 ± 0.3^c^	81.2 ± 0.5^b^	12.3 ± 0.7^c^
AT	18.7 ± 0.5^b^	79.1 ± 0.6^c^	15.5 ± 1.1^b^
MT	22.1 ± 1.1^a^	79.4 ± 1.2^c^	18.7 ± 1.3^a^

Note

The values are means ± standard deviation of three replicates. Means with different letter in a column differ significantly (*p* < .05).

Abbreviations: AT, autoclaving treatment; HMT, heat–moisture treatment; MT, microwave treatment; N, native proso millet starch.

### 3.2 Water holding capacity

The WHCs of proso millet starch after HMT, AT, and MT increased significantly (*p* < .05) to 172.66%, 191.63%, and 197.13%, respectively (Table 2). The increase in the WHC was mainly the result of starch gelatinization caused by thermal treatment. In addition, this indicates that a mass of bound water was produced by covalent bonding between hydroxyl groups and the starch molecular chains. The amount of resistant starch can be increased by gelatinization, and the increase in the number of hydrophilic hydroxyl groups on the outside of the glucose unit enables moisture absorption. According to Pinnavaia and Pizzirani (1998), the WHC of starch has a significant correlation with its gelatinization degree. Singh and Adedeji (2017) reported a similar trend for HMT proso millet starch, and an increase in AT samples from different botanical sources was reported by Hoover (2010).

**Table 2:** Water‐holding capacity properties native proso millet starch, HMT, AT, and MT starches

Type	Water‐holding capacity (%)
N	132.47 ± 1.93^d^
HMT	172.66 ± 2.26^c^
AT	191.63 ± 2.25^b^
MT	197.13 ± 2.42^a^

Note

The values are means ± standard deviation of three replicates. Means with different letter in a column differ significantly (*p* < .05).

Abbreviations: AT, autoclaving treatment; HMT, heat–moisture treatment; MT, microwave treatment; N, native proso millet starch.

### 3.3 Solubility and swelling power

**Figure 1:**
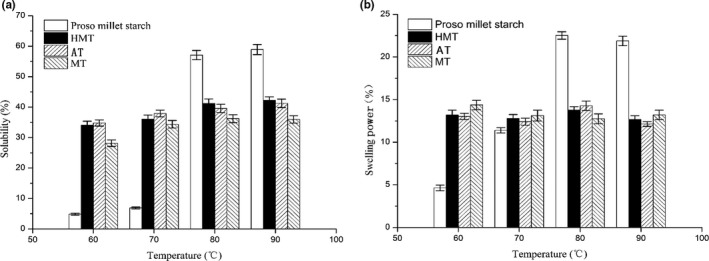
(a), The solubility of proso millet starch, HMT, AT, and MT starches at 60, 70, 80, and 90°C. (b), The swelling ability of proso millet starch, HMT, AT, and MT starches at 60, 70, 80, and 90°C. AT, autoclaving treatment; HMT, heat**–**moisture treatment; MT, microwave treatment

The solubility and swelling power of the native proso millet starch and modified samples are shown in Figure 1. The figure shows that the solubilities of all the samples increased when the temperature was increased to 60 and 70°C. The solubilities of the HMT, AT, and MT samples were clearly higher than that of native proso millet starch. The solubilities then decreased at 80 and 90°C, and the solubilities of the HMT, AT, and MT samples became lower than that of native proso millet starch. The treatment affects hydrogen bonding; and therefore, the structures became tighter and more ordered.

Heat–moisture treatment, AT, and MT decreased the swelling power of the native proso millet starch. The reduction in the swelling power of the starch after different thermal treatments can be attributed to reorganization of the amylose and amylopectin molecules and to additional amylose–amylose interactions, which restrict starch hydration. The increasing gelatinization temperature also explains the changes in the swelling power of the modified starch. According to previous research (Lawal & Adebowale, 2005; Zavareze & Dias, 2011), the decreased swelling power of HMT starch compared with that of native starch can be attributed to internal reordering of starch granules, which increases interactions between starch functional groups, and the formation of amylose–lipid complexes within starch granules.

### 3.4 Molecular weight distribution

The *M*_w_ of the heat‐treated samples were determined by HPSEC‐MALLS‐RI; the results are shown in Table 3. The *M*_w_ of the proso millet starch used in this work was 8.694 × 10^6^ g/mol, which is lower than previously reported values. This can be attributed to differences botanical origins of the starch, processing methods, and starch structures (Yoo & Jane, 2002; Zhang et al., 2014). In this study, thermal treatment of starch led to decreases in both the *M*_w_ and *R*_z_ values. HMT, AT, and MT decreased the *M*_w_ values, especially MT. The *M*_w_ values were 1.769 × 10^6^ g/mol after HMT, 7.886 × 10^5^ g/mol after AT, and 3.411 × 10^4^ g/mol after MT. These changes may be related to changes in the amylose content. Previous research showed that *M*_w_ decreases with increasing amylose content (Aberle, Burchard, Vorwerg, & Radosta, 1994; Fishman, Rodriguez, & Chau, 1996), and this is also consistent with the amylose content results. The results show that thermal treatment decreased the *M*_w_ of proso millet starch.

**Table 3:** MALLS parameters of native proso millet starch, HMT, AT, and MT starches^a^

Sample	*M*_w_ (g/mol)	*R*_z_ (nm)	PDI
N	8.694 ± 0.15 (×10^6^)	154.5 ± 0.05	1.243 ± 0.21
HMT	1.769 ± 0.01 (×10^6^)	120.3 ± 0.10	1.065 ± 0.01
AT	7.886 ± 0.04 (×10^5^)	94.1 ± 0.05	1.871 ± 0.10
MT	3.411 ± 0.02 (×10^4^)	38.2 ± 0.10	1.595 ± 0.09

Abbreviations: AT, autoclaving treatment; HMT, heat–moisture treatment; MT, microwave treatment; *M*_w_, weight‐average molecular weight; N, Native proso millet starch; PDI, polydispersity index; *R*_z_, z‐average radius of gyration.

a Molecular characteristics measurement by HPSEC‐MALS‐RI.

The z‐average radius of gyration (*R*_z_) refers to the theoretical probability of finding a molecule at a given distance from the center. The branch length and chain‐branching mode affect the *R*_z_ value (Jackson, 2010). The *R*_z_ value is therefore affected by the botanical origins of the starch. The high *R*_z_ of the proso millet used in this work can be attributed to high amylopectin content. The *R*_z_ value was decreased by thermal treatment; this result is consistent with previous research (Yang et al., 2017; Zeng, Ma, Kong, Gao, & Yu, 2015). In addition, the decreased *R*_z_ indicates a reduction in the branching degree.

The PDI is the ratio of the weight‐average molecular weight to the number‐average molecular weight; it is related to the molecular mass and polydispersity of the starch (Shin et al., 2009). The PDI values of the native proso millet starch and HMT starch were close to 1, which shows a narrow distribution and implies that the relative molecular mass distribution is uniform. However, the PDI values of AT and MT samples were greater than 1, which indicates a wider molecular weight distribution. For the HMT starch, the PDI was close to 1, which indicates that the starch was degraded during HMT, and molecular chains with a low degree of polymerization were formed. In contrast, the PDI values of the AT and MT starches increased to 1.871 and 1.595, respectively. This shows that AT and MT increased the polydispersity due to the molecular‐chain breakage. This suggests that thermal treatment led to formation of more short and branched chains in the starch system. The results show that thermal treatment alters the structure and properties of proso millet starch.

### 3.5 X‐ray diffraction

**Figure 2:**
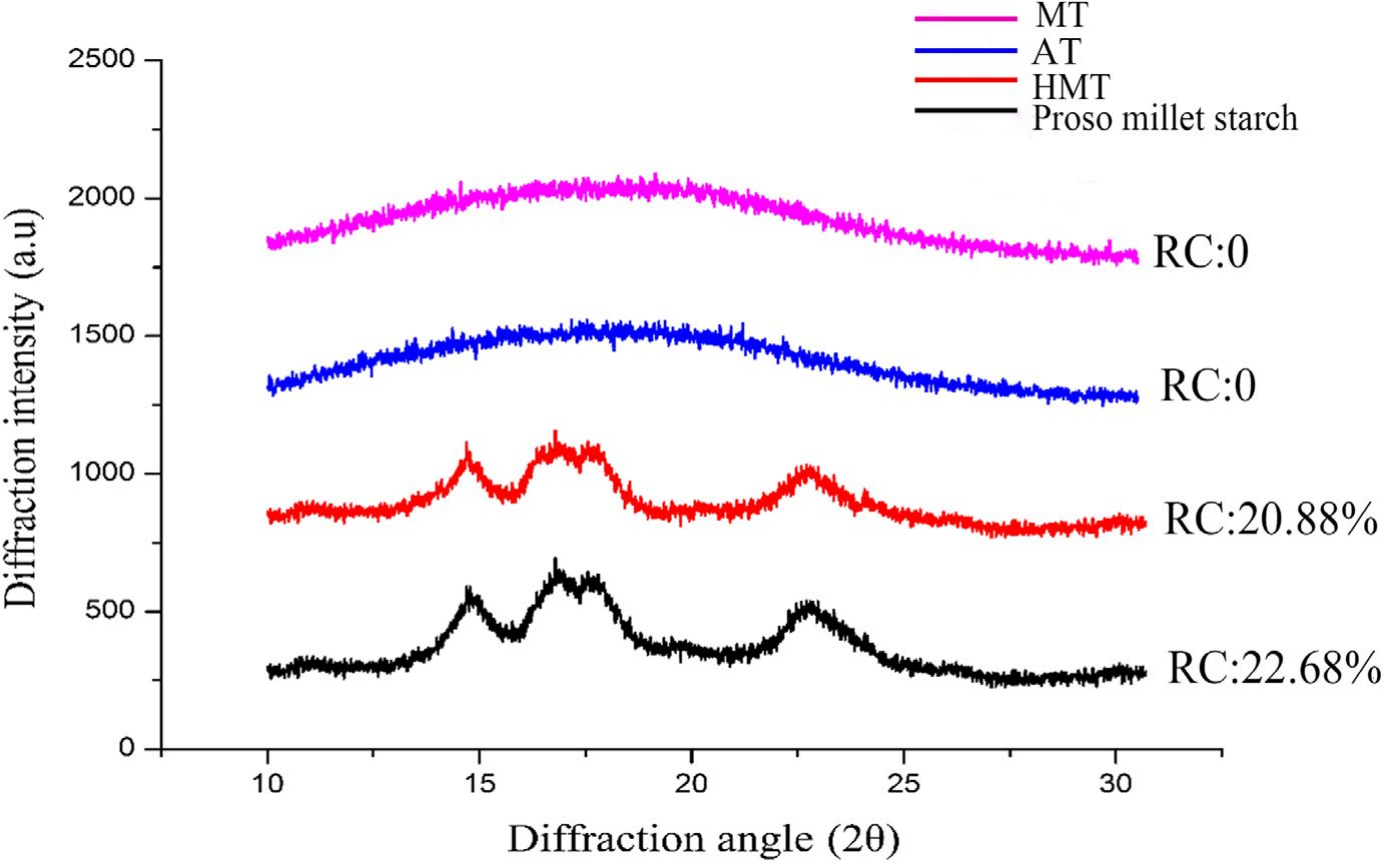
X‐ray diffraction patterns of proso millet starch, HMT, AT, and MT starches. AT, autoclaving treatment; HMT, heat**–**moisture treatment; MT, microwave treatment; RC: relative crystallinity

The XRD patterns and peak intensities of the samples are shown in Figure 2. The native proso millet starch and HMT sample gave an A‐type pattern, but no peaks were detected in the MT and AT sample patterns. The main peaks in the diffraction patterns of proso millet starch were observed at 2*θ* = 14.8°, 16.6°, 17.8°, 19.7°, and 23°. For the HMT sample, although the crystalline structure of the sample was still A type, its relative crystallinity decreased and the peaks became dispersed. This shows that HMT and AT destroyed the starch crystalline structure, which decreases the crystallinity. No peaks were detected in the pattern of the MT sample. This may be because microwave gelatinization completely destroys the starch crystal structure, and the molecular chains are broken. It has been reported that the vibrations caused by microwave nonthermal effects accelerate destruction of the semicrystalline growth ring structure of starch (Fan et al., 2014).

The HMT starch relative crystallinity (20.88%) was much lower than that of the native proso millet starch (22.68%). It has been reported that the starch crystallinity is inversely proportional to the amylose content. In this study, all three treatments increased the amylose content, which is consistent with previous results. The crystallinity is affected by crystal size, amylopectin content, amylopectin chain length, extent of interactions between double helices, and orientation of the double helices within the crystalline domains. Starch recrystallization is a complicated process, which involves conformational changes, chain alignment, crystal packing, and phase propagation (Sun, Gong, Li, & Xiong, 2014). Thermal treatment may have destroyed the starch crystalline structures. During thermal treatment, starch crystallites may have been disrupted or their orientation may have changed, as a result of partial or complete gelatinization and movement of double helices (Gunaratne & Hoover, 2002).

### 3.6 Differential scanning calorimetry

**Figure 3:**
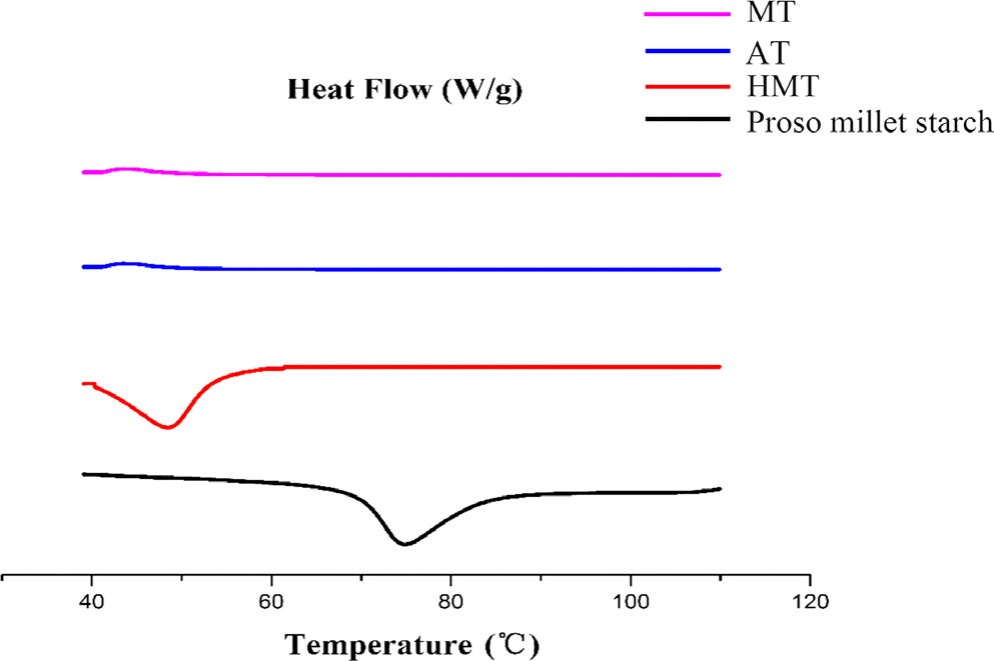
Differential scanning calorimetry thermograms of proso millet starch and HMT, AT, and MT starches. AT, autoclaving treatment; HMT, heat**–**moisture treatment; MT, microwave treatment

Thermograms of the samples are shown in Figure 3. It has been reported that the enthalpy values of gelatinized starch indicate melting of crystallites that were formed during gelatinization by association between adjacent double helices, and the endotherm peak is attributed to the melting of gelatinized amylopectins other than amylose (Krueger, Knutson, Inglett, & Walker, 2010). Paredes‐Lopez and Hernández‐Löpez (2010) suggested that during gelatinization process, numerous crystalline regions are formed. Gelatinized starches that have been reheated during DSC show lower enthalpies and crystallinities. The enthalpies of the modified starches can be differentiated due to the different thermal conductivities of the HMT, AT, and MT samples. In the case of HMT, heating caused breakage of the amylopectin branch of the α‐(1,6) bond, molecular degradation, and fracture or unwinding of the double helix structure; these give rise to disorganized starch molecular chains and curling. The hydroxyl groups in the molecular chains interact and ultimately form hydrogen bonds and new crystals with different stabilities. AT is based on HMT; excess water is added in the pressure field during gelatinization. During gelatinization, the temperature is gradually increased at constant pressure, and therefore, AT can cause changes to the crystal structure and imperfect crystal formation (Yang et al., 2016). The microwave heating of starch involves the dielectric heating effect of microwaves and the effect of electromagnetic polarization. Polar molecules in the starch particles in the microwave field rub together and collide with each other, and this generates a large amount of heat. Consequently, the starch grain temperature rises, which results in changes to the starch structure and physical and chemical properties (Fan et al., 2013; Lewandowicz, Jankowski, & Fornal, 2000). Microwave photon energy can affect the chemical bonds in the starch molecules and the arrangement of the electrons around a group. This changes the molecular conformation of the starch (Fan et al., 2014).

### 3.7 Pasting properties

The pasting properties of the samples are summarized in Table 4. All the modified starches showed significant changes (*p* < .05) in their pasting properties compared with those of native proso millet starch. The Rapid Visco Analysis results for the HMT, AT, and MT samples were lower (*p* < .05), and the PV, FV, BD, and SB values were higher than those of the native proso millet starch. The PV of the native proso millet starch (3,827 cP) was the highest (*p* < .05), and the PV of the HMT, AT, and MT samples was lower by varying degrees. This indicates that the interactions between starch chains slowed down, and hydrogen bond formation between chains decreased. Generally, the PV was positively related to the starch swelling power, which is consistent with the results of previous studies of swelling power. The BD values of HMT, AT, and MT decreased to 755, 1,085, and 838 cP, respectively. The BD value reflects the stability of a hot starch paste, and resistance to heat and shear. A decrease in the BD indicates that the starch grains have become stronger, which hinders swelling and rupture and gives good thermal stability after processing. Compared with that of native proso millet starch, the SB values of HMT, AT, and MT decreased to 267, 364, and 313 cP, respectively. The SB value reflects the stability and aging trend of a cold starch paste and is related to the degree of polymerization of amylose and the amylopectin structure. Amylose with a low polymerization degree and amylopectin with short outer chains can lead to cold starch paste stable retrogradation (Doutch et al., 2012; Hoover, 2010).

**Table 4:** Pasting properties of native proso millet starch, HMT, AT, and MT starches

Type	Peak Viscosity (cp)	Though Viscosity (cp)	Break down (cp)	Final Viscosity (cp)	Setback (cp)	Pasting temperature (°C)
N	3,827 ± 22^a^	2,122 ± 38^a^	1,705 ± 32^a^	2,876 ± 43^a^	754 ± 38^a^	76.03 ± 1.7^a^
HMT	1,483 ± 12^d^	728 ± 12^bc^	755 ± 14^d^	995 ± 25^b^	267 ± 12^d^	51.00 ± 2.1^b^
AT	1,846 ± 17^b^	761 ± 12^b^	1,085 ± 25^b^	1,125 ± 33^b^	364 ± 22^b^	50.15 ± 1.6^b^
MT	1,551 ± 14^c^	713 ± 11^c^	838 ± 17^c^	1,026 ± 33^b^	313 ± 20^c^	50.55 ± 1.3^b^

Note

The values are means ± standard deviation of three replicates. Means with different letter in a column differ significantly (*p* < .05).

Abbreviations: AT, autoclaving treatment; HMT, heat–moisture treatment; MT, microwave treatment; N, Native proso millet starch.

### 3.8 Scanning electron microscopy

**Figure 4:**
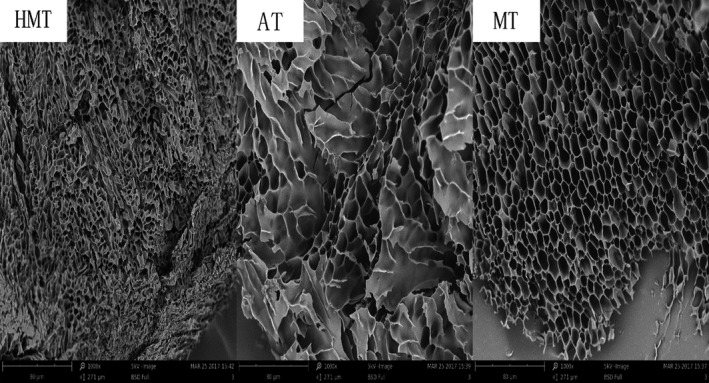
Scanning electron micrographs of HMT, AT, and MT starches gel**.** AT, autoclaving treatment; HMT, heat**–**moisture treatment; MT, microwave treatment

Scanning electron microscopy was used to investigate and confirm changes in the surface morphologies of the starch gels (Figure 4). Changes in the morphologies of starch granules are related to interactions between crystalline and amorphous regions in the starch (Wasserman et al., 2004). The microstructure of the gel obtained by HMT was a honeycomb with a thick stromal wall and pores of nonuniform size. The AT gel had more surface folds, and its stromal wall was thicker, its network structure looser, and pores larger than those of the HMT gel. The stromal wall of the MT gel was thin, and it had a uniform pore size and a neat regular network structure. The main reason for the differences among the gel microstructures is that different expansion and rupture spaces were provided for the starch granules during different heating processes. HMT, AT, and MT evidently affected the form and degree of agglomeration of granules. This is reasonable because of the partial gelatinization caused by moisture and the different thermal energies during HMT, AT, and MT. HMT leads to inconsistent swelling of the granules and the appearance of concavities on the surfaces; AT, which adds a pressure field to HMT, further increases the degree of gelatinization. In a microwave field, the effects of dielectric heating and electromagnetic polarization give rapid and regular starch gelatinization. The surface morphologies of the samples varied depending on the treatment. This suggests that HMT, AT, and MT destroyed the surface structures of the proso millet starch granules to different extents; this is in agreement with previously reported results (Zavareze, Storck, Castro, Schirmer, & Dias, 2010; Zhong et al., 2017).

## 4 CONCLUSIONS

This study explored the effects of HMT, AT, and MT on the physicochemical properties of proso millet starch. HMT, AT, and MT significantly affected the amylose and resistant starch contents, and the fractions of amylose and resistant starch; the values were highest after MT. The decreased *M*_w_ may be related to the amylose content, but there are many reasons for the molecular weight reduction, and further research is needed. HMT, AT, and MT affected the WHC, solubility, and swelling power of proso millet starch in varying degrees. A decreased swelling is desirable for some food applications such as noodle production.

Heat–moisture treatment decreased the relative crystallinity compared with that of native proso millet starch; AT and MT were more effective in reducing the structural recrystallization of the starch samples. The microstructure of a gel obtained by AT had a thicker stromal wall and looser network structure than those of a HMT gel. MT gave a thinner stromal wall, and the network structure was neater and more regular. The different microstructures may affect the physicochemical properties of proso millet starch to different degrees. RVA and DSC showed that the thermal properties of the samples improved after all three treatments, the setback and BD values decreased significantly, and the cold paste and hot paste stabilities of the samples improved. Further studies are needed to investigate the potential use of proso millet starch to improve the physicochemical and functional properties while increasing the nutritional value.

## CONFLICT OF INTEREST

The authors declare that they do not have any conflict of interest.

## ETHICAL APPROVAL

The study did not involve any human or animal testing.

## INFORMED CONSENT

Written informed consent was obtained from all study participants.
